# Analytical quality by design based on design space in reversed-phase-high performance liquid chromatography analysis for simultaneous estimation of metformin, linagliptin and empagliflozin

**DOI:** 10.1098/rsos.220215

**Published:** 2022-06-15

**Authors:** Aya A. Marie, Mohamed M. Salim, Amira H. Kamal, Sherin F. Hammad, Mahmoud M. Elkhoudary

**Affiliations:** ^1^ Department of Pharmaceutical Chemistry, Faculty of Pharmacy, Horus University-Egypt, New Damietta 34517, Egypt; ^2^ Department of Pharmaceutical Analytical Chemistry, Faculty of Pharmacy, Mansoura University, Mansoura 35516, Egypt; ^3^ Department of Pharmaceutical Analytical Chemistry, Faculty of Pharmacy, Tanta University, Tanta 31527, Egypt

**Keywords:** analytical quality by design, ATP, metformin, linagliptin, empagliflozin, human plasma

## Abstract

Employing the Quality by Design paradigm through this work helped conclude the method operable design region for optimizing the high performance liquid chromatography (HPLC) assay using Design of Experiments and response surface methodology to obtain a good resolution and determination of all analysed compounds and to achieve a suitable analysis time. A deep understanding of the quality target product profile, analytical target profile and risk assessment for parameters that affect the method performance led to developing an accurate, precise and cost-effective method. Quality risk management principles were applied for determining the critical method parameters affecting the simultaneous determination of metformin hydrochloride (MET), linagliptin (LIN) and empagliflozin (EMP) by reversed-phase HPLC . The ternary mixture was successfully resolved in 5 min with a linearity range of (0.1–600) µg ml^−1^ for MET and (0.05–50) µg ml^−1^ for LIN and EMP. The newly developed method was validated according to the International Council for Harmonization of Technical Requirements for Registration of Pharmaceuticals for Human Use guidelines. Good agreement was observed with the assay results of the reported UPLC one. To evaluate the greenness of the proposed method, an analytical Eco-Scale method was used.

## 1. Introduction

The 100 million Health Initiative in Egypt revealed that 2 million and 552 thousand people are likely to have diabetes, 10 million 358 thousand people with hypertension and 19 million and 561 thousand obese. They were referred to 302 referral centres nationwide for treatment [1].

Since managing type 2 diabetes milieus often needs combination therapy, using fixed-dose combination therapy led to relieving symptoms, slowing the progression of the illness, letting therapy be tailored to the individual, decreasing or preventing the requirement for hospitalization, decreasing healthcare costs [2,3].

These multicomponent preparations represent a challenge in developing a suitable validated analytical methodology applicable to their assay and quality control. Linagliptin (LIN), metformin hydrochloride (MET) and empagliflozin (EMP) are co-formulated in tablet dosage form Trijardy^®^ XR manufactured by Boehringer Ingelheim Pharmaceuticals, Inc US, which can be used in adults with type 2 diabetes. These drugs are the best choices for patients requiring glucose-lowering with multicomponent preparations [2]. Empagliflozin in the triple therapy mixture is added to decrease the risk of cardiovascular death in type 2 diabetes with cardiovascular diseases patients [4].

MET (electronic supplementary material, figure S1) is an oral antidiabetic that belongs to the biguanide class. It is the best choice for managing and treating type 2 diabetes [2]. It suppresses glucose production by the liver and reduces low-density lipoprotein, cholesterol and triglyceride levels [2]. LIN (electronic supplementary material, figure S1) is an inhibitor of dipeptidyl peptidase IV (DPP-4). It stimulates insulin release in a glucose-dependent way and decreases the glucagon levels in the blood [2]. EMP (electronic supplementary material, figure S1) is an inhibitor of the sodium–glucose cotransporter 2 (SGLT-2) responsible for 90% of glucose reabsorption. The inhibition effect of SGLT-2 decreases the blood glucose level by preventing the kidney reabsorbtion and thereby excreting glucose (i.e. blood sugar) via the urine [5].

Several analytical approaches were developed and reported for the determination of MET [6–8], LIN [9–11] and EMP [12–14] in single forms and combinations [15–21]. Two analytical methods were reported to simultaneously determine the triple therapy MET, LIN and EMP using ultra high performance liquid chromatography (UPLC) [22] and thin-layer chromatography densitometric methods [23]. Another method was recently proposed by S. Gurrala *et al*., where the study used the analytical Quality by Design (AQbD) paradigm and method operable design region (MODR) in optimization of a high performance liquid chromatography (HPLC) method for the simultaneous determination of the MET, LIN and EMP [24]. However, the article included many downsides that may have affected the validity and integrity of the applied AQbD and MODR procedures, e.g. methodology of selection of Taguchi design significant factors, very low s.d. and coefficient of variation per cent values that did not reflect the high variability in the centre level points and design points of the Central Composite Design (CCD), and MODR selection criteria were neither mentioned nor matched the mathematical proposed criteria for method optimization.

Thus, developing an accurate, rapid and precise method for analysing antidiabetic drugs becomes necessary, especially for fixed-dose combinations. The thrust towards simple, rapid and environmentally benign analyses for these complex mixtures mandates exploring univariate and multivariate techniques to extract relevant information from the analysis data. Numerous analytical methods used the Design of Experiments (DOE) to develop and/or optimize analytical methods [25,26].

In the previous, the chromatographic analytical methods were optimized based on the one factor at a time method (OFAT). This method depends on changing a single factor affecting the method performance and keeping other parameters constant. So, these procedures require many experiments, and once developed, the process will still require extra work when validated and extended for biological fluids determination [27].

Opposing the OFAT approach, Quality by Design (QbD) is a proactive and risk assessment technique for method development that depends on recognizing and minimalizing causes of divergence that may affect the robustness [28,29]. The present work depends on the recognition of the quality target product profile (QTPP), then the analytical target profile (ATP), and risk assessment for parameters that can significantly affect the method performance [30]. Depending on the International Council for Harmonization of Technical Requirements for Registration of Pharmaceuticals for Human Use (ICH) Q8(R2) and ICH Q9 [31,32], the ATP is preferable for use for specifying the MODR, intending to lessen the number of out of trend (OOT) and out of specification (OOS) results.

DOE uses multivariate statistical methods. Such methods have advantages, such as decreasing the number of the experiments, decreasing consumption of reagents, less laboratory work, using mathematical models and permitting estimation of relevance and the significance of the effects between many trivial factors to qualify the vital few ones [33]. If significant interaction occurs between parameters, the optimum chromatographic conditions obtained by the univariate or OFAT methods will differ from the exact conditions developed by the multivariate methods. The factor's interaction effect is directly proportional to this difference found between multivariate and univariate results as the effect of one factor may be based on other factors. By contrast, in a multivariate approach, the levels of different factors are simultaneously altered to determine the interaction effects of different factors affecting method performance [34]. The ATP was to develop a highly sensitive, robust, rapid and green reversed-phase HPLC (RP-HPLC) method that can separate the three antidiabetic drugs MET, LIN and EMP. Defining the objectives, critical method parameters (CMPs), and critical quality attributes (CQAs) is crucial in developing methods that achieve ATP aims. The developed and optimized QbD method was validated according to ICH Q2(R1), and successfully applied to the determination of MET, LIN and EMP in their pharmaceutical preparations and to broaden the application scope to human plasma.

The present work aims to discuss the outcomes of using AQbD paradigms for developing and optimizing a green RP-HPLC technique for the simultaneous quantitation of the three co-formulated antidiabetic drugs.

## 2. Experimental

### 2.1. Active pharmaceutical ingredients and excipients

MET (99.00%) and LIN (99.70%) were friendly provided by Ateco for pharmaceutical industries (Mubarak Industrial Zone, Quesna - Menoufia – Egypt). EMP (99.10%) and dosage form excipients: polyethylene oxide, hypromellose, magnesium stearate, hydroxypropyl cellulose, talc, titanium dioxide, arginine, carnauba wax and shellac glaze all were friendly obtained from Sigma for pharmaceutical industries (Mubarak Industrial Zone, Quesna - Menoufia – Egypt).

Dosage form Gliptalina^®^ (Rameda, Egypt), batch no: 190120, containing 2.5 mg of LIN and 500 mg of MET and Empagliform^®^ (Hikma, Egypt), batch no: 00319, containing 12.5 mg of EMP and 500 mg of MET were provided from the local market.

### 2.2. Reagents

Methanol (HPLC grade-Fisher, UK); orthophosphoric acid of analytical grade (Sigma-Aldrich, Germany); potassium dihydrogen phosphate (Inter. Trade Co., Japan); and triethylamine (TEA; HPLC grade-Oxford Laboratory, UK). Human plasma was obtained from the blood bank of Tanta University Hospital (Tanta – El-Gharbia – Egypt).

### 2.3. Apparatus and high performance liquid chromatography software

Dionex UltiMate 3000 RS system (Thermo Scientific, Dionex, Sunnyvale, CA, USA), equipped with a Quaternary RS pump, thermostated RS column compartment, RS diode array detector (DAD), and RS auto-sampler injector. The data acquisition was completed by ChromeleonR 7.1 software. Hettich Centrifuge (Tuttlingen, Germany) and Vortex (A & E, UK). A HANNA pH-meter (USA). DOE, response surfaces, and other diagrams were performed using Design-Expert^®^ v. 11 software [35].

### 2.4. Chromatographic conditions

The separation mobile phase consists of 0.043 M potassium dihydrogen orthophosphate buffer premixed with 0.05%v/v TEA (buffer pH 3.79 adjusted using orthophosphoric acid): methanol (34.4:65.6, v/v) was used. A Thermo Hypersil octa decyl silane (250 mm × 4.6 mm, 5 µm) column, the column temperature was 40°C, and the flow rate was 1 ml min^−1^. The DAD detector was set at a range of (210–350) nm, and 225 nm was selected as an optimum wavelength. The injection volume was 10 µl.

### 2.5. Preparation of standard solutions (stock/working)

Stock solutions (1000 µg ml^−1^) of MET, LIN and EMP were prepared in methanol, while working solutions were prepared freshly by the suitable dilution of the stock solutions with the mobile phase to get 100 µg ml^−1^ of MET, 50 µg ml^−1^ of both LIN and EMP. The solutions were stable when kept at 4°C based on 98% recovery.

### 2.6. Preparation of plasma samples

The blank human plasma sample was thawed for 1 h before the analysis at room temperature. Then the samples were vortexed at 4000 r.p.m. for 30 s to ensure complete mixing of the sample's contents. One hundred microlitres of blank plasma sample were transferred into centrifuge tubes, different volumes were added from 50 µg ml^−1^ working standard solutions of each of the three drugs, and the above solutions were completed to 5 ml with methanol for protein precipitation and vortexed twice to mix at 2000 r.p.m. for 30 s. These samples were centrifuged at 4000 r.p.m. for 30 min, 1 ml of each clear supernatant was transferred into a 5 ml volumetric flask and diluted up to 5 ml with mobile phase, and 10 µl from each solution was injected at the previously mentioned chromatographic conditions.

### 2.7. Construction of calibration curves

#### 2.7.1. Calibration curves for standard solutions

Different aliquots of MET, EMP and LIN working standard solutions were taken in 10 ml volumetric flasks and diluted with mobile phase to get solutions in the concentration range 0.1–600 µg ml^−1^ MET and 0.05–50 µg ml^−1^ EMP and LIN. Ten microlitres of each solution was injected under the optimal chromatographic conditions. Calibration curves were constructed for each drug by plotting the average peak area (triple) versus drug concentrations (µg ml^−1^), and their regression equations were computed.

#### 2.7.2. Matrix matched calibration in spiked human plasma

Calibration curves were constructed for three drugs by plotting the average peak area (triple) against the drug concentration in spiked human plasma samples covering the range of 0.1–2 µg ml^−1^ for MET and 0.05–2 µg ml^−1^ for both LIN and EMP.

### 2.8. Analytical Quality by Design paradigm

#### 2.8.1. Outline the quality target product profile, analytical target profile, and identification of critical quality attributes

In the AQbD approach, the determination and outline of the QTPP of the final pharmaceutical product was the first step. Then the ATP was determined depending on the previously recognized QTPP. Further on, the CQAs were recognized depending on literature review and preliminary trials.

#### 2.8.2. Analytical Quality by Design-based risk assessment

Risk assessment was performed to identify CQAs that could affect the performance of the analytical method. Risk analysis tools such as Ishikawa or fishbone diagram can aid in recognizing and controlling possible quality problems. Quality risk management principles were performed for defining the impact of different CMPs on the CQAs.

An Ishikawa or fishbone diagram was drawn for a risk assessment for the factors that majorly affect the RP-HPLC method's performance (CMPs), as shown in the electronic supplementary material, figure S2.

Factors affecting different chromatographic procedure aspects (e.g. system, column, mobile phase, elution, injection and detection) were checked. Depending on the previously specified ATP, the measured responses were assigned as CQAs (number of theoretical plates (NTP) of LIN, Asym of LIN, resolution-1 (Rs-1) between (MET-LIN) and resolution-2 (Rs-2) between (LIN-EMP)) affecting the final performance of the analytical method. Subsequently, preliminary trials demonstrated that three of the tested parameters (CMPs): percentage of organic modifier (%MeOH), buffer pH and buffer concentration affected the overall chromatographic performance and the separation of the studied compounds. These parameters were used for the optimization phase.

#### 2.8.3. Analytical Quality by Design-based method optimization using response surface design

The concluded significant CMPs obtained from the preliminary studies results were optimized using response surface methodology to determine the optimum levels of each with consideration of possible quadratic effects. A three-factors (%MeOH, buffer pH and buffer concentration) and three-levels rotatable CCD were developed. A total of 20 experimental runs: 14 non-centre points (eight factorial runs + six axial points (*α* = 1.68)) and six centre points to consider the experimental errors. These experiments helped to build a model that could optimize the three selected CMPs relying on the results of four CQAs named: NTP of LIN, Asym of LIN, Rs-1 between (MET-LIN) and Rs-2 between (LIN-EMP).

#### 2.8.4. Selection of the method operable design region

The MODR was established depending on the regression models for each CQA. All stated ATP criteria were satisfied at a specified risk level within this region. The selected CQAs were predicted and plotted according to their tolerance interval (TI) using the acceptable delta (d) and sigma (s) values and with a significance level of (*α*) = 0.05 and with an outcome proportion that achieved the TI specifications of 0.95 (one-sided). The domain of the experimental space was where TI criteria intersect was considered the MODR of the developed HPLC procedures. Software-based models applying derringer's desirability algorithm suggested the optimum levels of each parameter based on the defined optimization criteria. These levels were then validated and adopted.

### 2.9. Method validation

The developed AQbD method was validated according to ICH Q2R1 guidelines [36]. The proposed HPLC method was applied over the ranges of (0.1–600 µg ml^−1^ for MET, and 0.05–50 µg ml^−1^ for LIN and EMP). The quantitation limit (LOQ) and detection limit (LOD) were calculated referring to equations (2.1) and (2.2):

2.1 $$\mathrm{LOQ}=10S_{a}/b,$$

and

2.2 $$\mathrm{LOD}=3.3S_{a}/b,$$

where *S*_a_ is the standard deviation of the *y*-intercept of regression lines and *b* is the slope.

The method's accuracy was evaluated by calculating the mean per cent recoveries of three determinations of the studied drugs at three different concentration levels within the linearity range. To determine inter-day and intra-day precision, three different concentrations of each drug were assessed on three consecutive occasions between one day and three successive days.

Method robustness in AQbD-developed analytical methods can be done for justification and confirmation of MODR. The results were compared with that concluded from MODR to assess the agreement and disagreement points.

The stability of the studied drugs was assessed; MET was stable for one month, while LIN and EMP stock solutions were stable for two weeks at 4°C in the refrigerator.

### 2.10. Analytical eco-scale approach for assessment of the method greenness

The analytical eco-scale method was applied to assess the greenness of the proposed method. It depends on the calculation of the penalty points of an analytical process. An ideal green analysis is characterized by minimizing the use of energy, hazardous substances and waste generation [37]. There were many recent applications of analytical eco-scale in liquid chromatography methods [38–41].

In this approach, the ‘ideal’ analysis has a total score of 100. For each parameter that varies from ‘the ideal value,’ penalty points are calculated, decreasing the method score. Thus, the higher the score, the greener. The analytical eco-scale total score is shown in equation (2.3) [37]:

2.3 analytical eco−scale=100−sum of total penalty points

### 2.11. Application to laboratory prepared tablet

Trijardy^®^ XR tablet dosage form composed of 1000 mg MET, 25 mg EMP and 5 mg LIN [41] is not present in the local Egyptian market, so the laboratory prepared tablets. Referring to the preparation of laboratory prepared tablet [42] the formula per tablet was prepared by weighing 1000 mg of MET + 5 mg of LIN + 25 mg EMP + 500 mg polyethylene oxide + 500 mg hypromellose + 20 mg magnesium stearate + 20 mg hydroxyl propyl cellulose + 10 mg titanium dioxide + 10 mg arginine + 10 mg shellac glaze. The weight of powdered tablets containing 500 mg MET, 2.5 mg LIN and 12.5 mg EMP was taken into a 100 ml volumetric flask; and dissolved in 70 ml methanol. After 10 min sonication, the solution was left at room temperature then the volume was completed to the mark with methanol. Then the solution was filtered, the residue was washed, and a certain aliquot of the filtrate was taken. Suitable dilutions using the mobile phase were made to obtain a final concentration of 500 µg ml^−1^ MET, 2.5 µg ml^−1^ LIN and 12.5 µg ml^−1^ EMP.

### 2.12. Application to local market dosage forms: (binary combinations)

#### 2.12.1. Gliptalina®

Ten tablets, Gliptalina^®^, were weighed and finely powdered. A weight of powdered tablets equivalent to 2.5 mg of LIN and 500 mg of MET was weighed and transferred to a 100 ml volumetric flask. The drug was dissolved with 70 ml methanol, sonicated for 15 min, left at room temperature and completed to the mark using methanol. The solution was filtered, the residue was washed, and serial dilutions using the mobile phase were carried out to obtain different drug concentrations.

#### 2.12.2. Empagliform®

Ten tablets^®^, Empagliform, were weighed and finely powdered. A weight of powdered tablets equivalent to 12.5 mg of EMP and 500 mg of MET was weighed and transferred to a 100 ml volumetric flask. Then, the same preparation steps were followed as in Gliptalina^®^.

## 3. Results and discussion

The following steps were followed for the development and optimization of the proposed RP-HPLC method using AQbD; then merits of this method were used to obtain the MODR and optimum HPLC method parameters.

### 3.1. Analytical Quality-by-Design paradigm

#### 3.1.1. Outline the quality target product profile, analytical target profile, and identification of critical quality attributes

Dosage form, route of administration, stability and delivery system must be considered to define the QTPP [31,43]. Also, the ATP was recognized depending on the defined QTPP, which involved a highly efficient RP-HPLC analytical method for good determination of all studied drugs within an acceptable range (98–102) per cent, a reasonable specificity (no interference from excipients), sharp symmetrical peaks with a suitable retention time. Further on, the critical quality attributes were recognized depending on preliminary trials and literature reviews.

#### 3.1.2. Analytical Quality-by-Design-based risk assessment

The risk assessment helps define the CQAs that affect the performance of the analytical method. Considering previous scientific knowledge and preliminary tests, a risk assessment study that relied on the Ishikawa diagram (electronic supplementary material, figure S2) was achieved. As followed in many HPLC development literature, the number one priority for the researcher is to achieve the best possible analysed components' separation with the highest sensitivity and lowest run time.

Preliminary trials were carried out by trying different columns, organic modifiers, aqueous phase (water and buffers), proportions of the mobile phase and different flow rates. The results suggested that problems in peak asymmetry as well as the resolution and sensitivity needed to be considered. Standing on our work experience; peak asymmetry is strongly affected by the per cent of organic modifier, column temperature and pH of the buffer, which affects the silanol ionization (at lower pH of the liquids (pH: 3), silanol ionization is suppressed) [44]. On the other hand, NTP can be affected by the flow rate, column temperature, the viscosity of the mobile phase and the molecular weight of the analyte. Resolution is a crucial HPLC performance indicator usually assessed by how quickly and utterly different target components in a sample were separated as they passed through a column. Resolution can be affected by column stationary phase, column length, particle size, column temperature, buffer pH, mobile phase solvent(s) and ionization (polarity) of the analyte affected by the mobile phase's different pH. Peak symmetry affects column efficiency and, therefore, resolution.

A DAD was used for detection; the wavelength at 225 nm was chosen efficiently based on the UV signal intensity of each of the analysed components and its ratio in the dosage form. The wavelength achieved the highest signal intensity for LIN (dosage form ratio 1: 5: 200, LIN: EMP: MET; respectively) and relatively lower signal intensity for MET.

The asymmetry of the marginally tailed peaks as LIN (previously studied) and, to a lesser extent, MET (both are NH_2_ rich compounds) was correlated with the interaction with ionized silanol groups. The addition of a low concentration cationic pairing agent (e.g. TEA) masked the free silanol groups with the ethyl groups and C18 interactions hence improving peak asymmetry and peak shape of both LIN and MET. The concentration of 0.05% v/v produced the optimum tailing improvement with no further enhancement with increasing its concentration. The optimization of TEA concentration was confined to the previous trials to optimize the critical qualified parameters %MeOH, buffer pH and concentration.

The preliminary studies revealed that %MeOH, buffer pH, and buffer concentration were considered the most influential factors in most of the responses (CQAs). System suitability parameters and overall chromatographic analytical method performance needed further optimization owing to the different influences orientation of each parameter on the selected response model. To a lesser extent, temperature and flow rate were significant. So, their levels were held constant and were 1 ml min^−1^ (favoured for minimal mobile phase consumption with a suggestion to add an ion-pairing agent to compensate for the asymmetry values deterioration) and 40°C, respectively. Moreover, the previously noted important CQAs from preliminary studies (asymmetry, resolution, NTP) were used as the measured responses for optimization.

#### 3.1.3. Method operable design region and optimization using response surface design

AQbD aims to identify the MODR, which is the region of CMPs that meet the CQAs. In other words, MODR is a multidimensional combination and interaction of variables and process parameters demonstrated to provide quality assurance (ICH Q8(R2), 2009). The initial knowledge space was explored using the DOE strategy. From this knowledge space, we identified a design region where all the specifications mentioned in the ATP are fulfiled at a specified risk level (ICH Q8(R2), 2009). CCD with 20 runs was performed to evaluate the influences of the qualified parameters on the selected attributes (electronic supplementary material, table S1).

**Figure 1.**
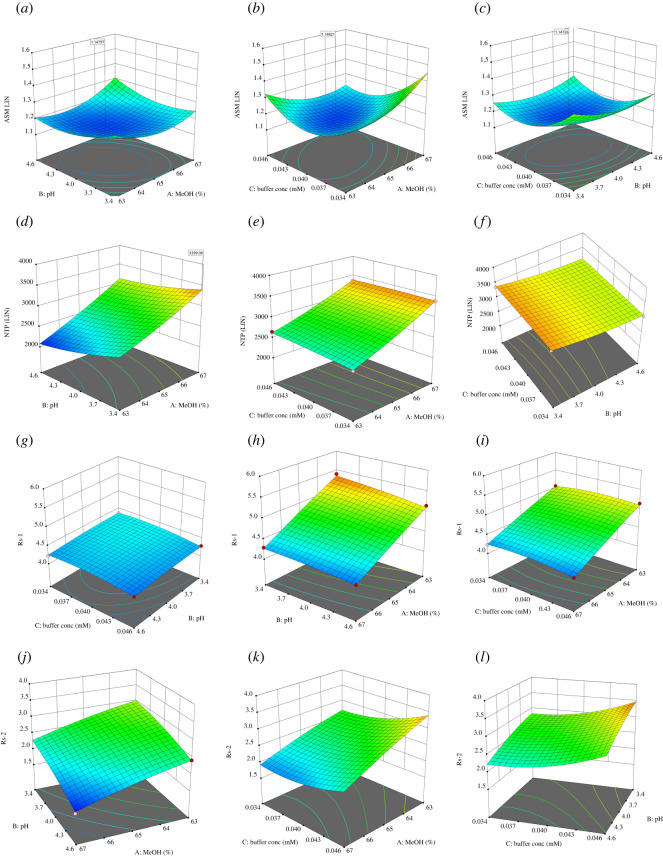
Response surfaces CCD for factor interaction. (*a*–*c*) Asym LIN; (*d*–*f*) NTP LIN; (*g*–*i*) Rs-1(MET-LIN); and (*j-l*) Rs-2 (LIN-EMP).

With the aid of the design tools, the polynomial equations and the relationship plots demonstrated the influences and interaction between the variables. For the observed responses, three-dimensional response surfaces showing the influences of the interaction of factors on the dependent responses were adopted, and an example is presented in figure 1.

Coefficients of each parameter were obtained after fitting the selected parameters following the ANOVA test for the experimental runs' data (table 1). All the developed models were quadratic, and the quadratic relationships were represented by higher-order terms (*x*^2^), where variables behaved nonlinearly.

**Table 1.** Coefficients and ANOVA statistical analysis for the three studied factors of the optimization design. (M, metformin; L, linagliptin; E, empagliflozin; Asym, asymmetry; *t*_R_, retention time; NTP, number of theoretical plates; Rs, resolution; A, %MeOH; B, buffer pH; C, buffer concentration; AB, %MeOH-buffer pH interaction; AC, %MeOH-buffer concentration interaction; A², B² and C², the quadratic terms of the corresponding factors.)

	A	B	C	AB	AC	A²	B²	C²
Rs 1	−0.47	−0.10		0.04				−0.07
*p*-values	<0.0001	<0.0001		0.0403				0.0004
Rs 2	−0.47	−0.32	0.27		−0.09	−0.05	−0.08	0.18
*p*-values	<0.0001	<0.0001	<0.0001		0.0167	0.024	0.013	<0.0001
NTP (LIN)	464.77	−181.50	38.88	49.71		41.86	59.91	
*p*-values	<0.0001	<0.0001	0.0241	0.034		0.0117	0.0059	
ASM LIN	0.02		−0.04	0.04	−0.07	0.07	0.05	0.10
*p*-values	0.0129		0.0002	0.0009	<0.0001	<0.0001	<0.0001	<0.0001

All the models showed high *R***^2^** and adjusted *R***^2^** values and insignificant lack-of-fit relative to pure error values, where all indicated good model fitting (electronic supplementary material, table S1).

Inspection of the developed model coefficients (table 2), as well as three-dimensional response surfaces (figure 1*a–l*) and contour plots, indicated the following.

**Table 2.** Regression analysis results for determination of MET, LIN and EMP in using the proposed HPLC method. (*a*, intercept; *b*, slope; *r*, correlation coefficient; *S*_a_, standard deviation of intercept; *S*_b_, standard deviation of slope; *S*_y/x_, residual standard deviation; LOD, limit of detection; LOQ, limit of quantitation.)

parameters	MET	EMP	LIN
concentration range (µg ml^−1^)	0.1–600	0.05–50	0.05–50
*R*	0.9999	0.9999	0.9999
*a*	0.077	0.085	0.198
*b*	0.387	0.330	0.923
*S*_a_	0.005	0.002	0.005
*S*_b_	2.329 × 10^−5^	8.700 × 10^−5^	2.61 × 10^−4^
*S*_(y/x)_	0.013	0.004	0.012
LOD	0.043	0.017	0.018
LOQ	0.130	0.051	0.054

**For Asym LIN** (figure 1*a*–*c*): all the surface plots showed symmetrically curved surfaces indicating the significance of quadratic terms owing to nonlinear parameter interactions. Values close to 1.1 were predicted when %MeOH (65%), buffer pH (3.98), buffer concentration (0.042 M) were used.

**For NTP (LIN)** (figure 1*d*–*f*): all the surface plots showed gradient planes with a slight curvature. Values close to 3400 were predicted when %MeOH (67%), buffer pH (3.40), buffer concentration (0.034–0.046 M) were used.

**For Rs-1 MET-LIN** (figure 1*g*–*i*): all the surface plots showed gradient planes with a slight curvature. Values close to 4.1 were predicted when %MeOH (67%), buffer pH (4.60), buffer concentration (0.034–0.046 M) were used.

**For Rs-2 LIN-EMP** (figure 1*j*–*l*): the AB surface plot showed a gradient plane with a slight curvature, while AC and BC surface plots showed a slightly asymmetric curved surface. Values slightly above 2 were predicted when %MeOH (66.5%), buffer pH (4.30), buffer concentration (0.041 M) were used.

To summarize the optimization results, %MeOH (factor A) again had the most prominent effect on all responses, considering the need to counterweight its level based on the optimization criteria. At the same time, buffer concentration (C) had the mildest effect on most of the modelled responses. Optimization criteria would help to select the buffer pH owing to the variability of the level that fits the needs of each modelled response.

The design region, shown in the electronic supplementary material, figure S3 (overlay plots), was created by performing a series of limitations (min, max) that achieve ATP with Rs-1 below 5, Rs-2 more than 1.6, NTP LIN more than 2500, and Asym LIN more than 0.8 with an outcome proportion that achieves the tolerance interval specifications of 0.95 (one-sided) as indicated in the electronic supplementary material, table S1.

The following criteria were depicted to optimize the different responses for optimum chromatographic performance within the predetermined MODR: minimize Rs-1 and Asym (LIN) and maximize Rs-2 and NTP (LIN). One solution was provided using Derringer's desirability algorithm (electronic supplementary material, figure S4) with the following parameter levels: %MeOH (65.6%), buffer pH (3.79), buffer concentration (0.043 M) with a desirability value of 0.531 and expected attribute values of Asym (LIN) (1.16), NTP (LIN) (2922), Rs-1 (4.70) and Rs-2 (2.68) as shown in the electronic supplementary material, figure S5(*a*–*c*). These proposed optimum conditions were tested, and the observed values were Asym (LIN) (1.18), NTP (LIN) (2907), Rs-1 (4.76) and Rs-2 (2.62). The results obtained practically were compared to the predicted values to examine the model predictability, and all the results were acceptable with very low prediction error.

Finally, the mobile phase was (65.6:34.4) MeOH: phosphate buffer (0.043 M) pH (3.79) with (0.05 v/v%) TEA. The flow rate used was 1 ml min^−1^, the column temperature was 40°C, and the DAD was used at 225 nm to detect the three drugs. System suitability parameters at optimum chromatographic conditions are shown in the electronic supplementary material, table S2.

Generally, there was a respectable difference between the total understanding of the chromatographic performance and effect of each parameter, its interaction, and nonlinear behaviour during the application of AQbD. Over and above the ability to determine space of acceptable analysis parameter levels that ensure repeatability of the developed method and lower failure probability, we can highlight the following merits.

Parameters (e.g. %MeOH, buffer pH and concentration) that showed a nonlinear response in AQbD optimization could only be optimized to the unit fractions using response surface methods (quadratic term representation). Otherwise, many trials would have to be made with tedious time and money consuming above and below specific parameters and levels to reach closer results to AQbD using OFAT optimization.

Parameter optimization based on selected criteria focused on analysis needs and the importance of each attribute model saved time and helped reach analysis goals as planned through AQbD and MODR.

### 3.2. Method validation

#### 3.2.1. Linearity and range

Linearity ranges were 0.1–600 µg ml^−1^ for MET and 0.05–50 µg ml^−1^ for LIN and EMP in pure form. Moreover, the range was 0.05–2 µg ml^−1^ for both LIN and EMP and from 0.1–2 µg ml^−1^ for MET in spiked plasma samples. Regression analysis was computed for the three studied drugs, as shown in table 2. The results indicate good linearity for three drugs, balanced residuals distribution, and (*r*^2^ > 0.999).

#### 3.2.2. Limits of detection and quantitation

As per the proposed analysis method, the values for LOD for the three drugs in pure form were 0.043, 0.018 and 0.017 µg ml^−1^ for MET, LIN and EMP respectively, while LOQ values were 0.130, 0.054 and 0.051 µg ml^−1^ for MET, LIN and EMP respectively. These limits were calculated for spiked plasma samples, 0.017, 0.013 and 0.007 µg ml^−1^ for MET, LIN and EMP respectively, for LOD, while LOQ values were 0.052, 0.040 and 0.021 µg ml^−1^ for MET, LIN and EMP respectively.

#### 3.2.3. Accuracy ‘trueness’

The method's accuracy was evaluated by calculating the mean per cent recoveries of three determinations of the three studied drugs at three different concentrations inside the linearity range. All calculated mean per cent recoveries of the three studied drugs were found to be within compendial tolerance (98–102%), as shown in the electronic supplementary material, table S3.

#### 3.2.4. Precision

Precision results were assessed by calculating (% RSD), All calculated (% RSD) values were (<2) as shown in the electronic supplementary material, table S4; indicating sufficient repeatability of the developed QbD method.

#### 3.2.5. Robustness

As per ICH guidelines [36], the robustness is a degree of its ability to persist unaffected by small but deliberate changes in analytical method parameters. Using the AQbD paradigm and building MODR helped in building method robustness and ruggedness before validation where MODR itself is the region of CMPs that meet CQAs. This can be considered as a key difference between OFAT and AQbD paradigms and represents the dissimilarity between traditional and modern method development procedures.

A factorial design of 13 runs with five factors, two levels (−1, +1) that were based on uncertainties accompanied these factors and 13 experiments were used in robustness testing (electronic supplementary material, table S5). Small changes in previously optimized factors (CMPs) were studied and the resulting pareto charts were inspected for significant factors and it was obvious that all the studied CMPs showed non-significant effects in the tested levels on the pre-selected responses (CQAs) in addition to the peak area of all the studied components (electronic supplementary material, figure S6(a–e)). These results indicated good stability of the developed method to small changes in its parameters as well as acceptable chromatographic performance. However, studying the t-value limit revealed that the flow was marginally significant to both Rs-2 and NTP LIN. Considering the quite small t-value of flow rate compared to Bonferroni limit we can overcome this consideration and consider flow rate as a non-significant (robust) chromatographic parameter.

There is no doubt that the results from the multivariate study and the predetermined MODR were more informative and enabled the determination of a wider range of robust and rugged method performance than the univariate one, with the merit of considering factor interactions that contribute to chromatographic performance.

#### 3.2.6. Specificity

**Figure 2.**
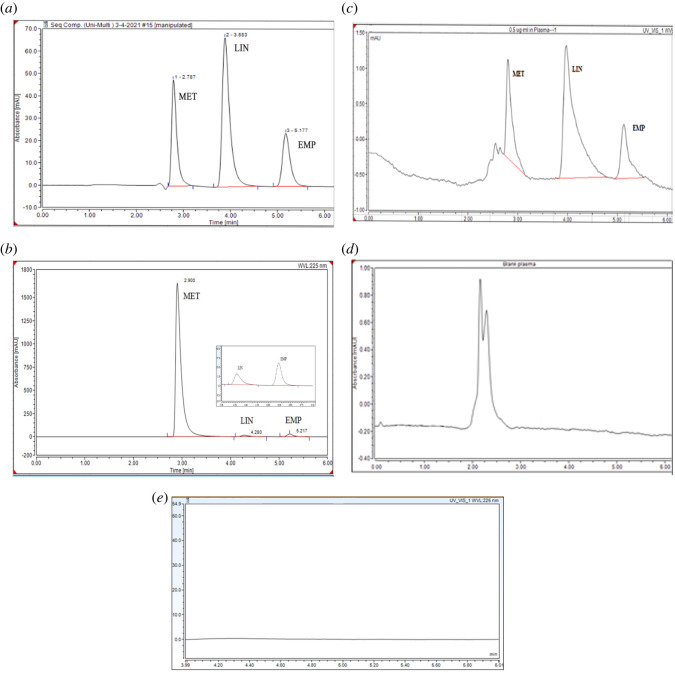
Chromatograms showing (*a*) the separation of MET, LIN and EMP in laboratory prepared mixture of 20 µg ml^−1^ using AQbD paradigm; (*b*) laboratory prepared tablets in the ratio 200 : 1 : 5 for MET : LIN : EMP respectively; (*c*) spiked plasma samples with 0.5 µg ml^−1^ of MET, LIN and EMP; (*d*) blank (non-spiked) plasma sample; and (*e*) blank mobile phase.

The developed and optimized method is specific based on ICH guidelines [36], as it was unaffected by the presence of excipients of dosage form. This was proved by comparing the chromatogram of laboratory tablets containing all probable excipients with the chromatogram of synthetic mixtures of the standard pure drugs of the same concentrations of the three drugs at the optimal chromatographic conditions as illustrated in figure 2*a,b*. Specificity was also proved by that the optimized method was unaffected by the presence of biological matrices of plasma (figure 2*c*,*d*).

### 3.3. Assay of synthetic mixture

Three mixtures of the three pure drugs were prepared in the ratio (200 : 1 : 5) MET : LIN : EMP, and injected triplicate using the optimal conditions of the proposed RP-HPLC method. Results as shown in the electronic supplementary material, table S6.

### 3.4. Assay of prepared tablet dosage form

The developed and optimized HPLC AQbD approach was used for the simultaneous identification and estimation of MET, LIN and EMP in their tablets in the ratio (200 : 1 : 5) (MET : LIN : EMP). The obtained results were satisfactory for each drug with good agreement with the labelled claim (figure 2*b*) and (table 3).

**Table 3.** Comparison between the assay of simulated tablets using the proposed HPLC method and reported UPLC.

drugs	proposed method			reported method [22]		
	%recovery MET	%recovery LIN	%recovery EMP	%recovery MET	%recovery LIN	%recovery EMP
mean (Ẋ)	100.974	99.326	100.739	100.778	99.657	99.842
*S*	0.079	0.097	0.053	0.557	0.719	0.918
%RSD	0.079	0.098	0.053	0.552	0.722	0.919
*T*_cal_	0.608	0.791	1.689	*T*_tab_		2.37
*F*_cal_	0.020	0.018	0.003	*F*_tab_		5.79

Using *t*-tests and *F*-tests at 95% confidence level statistical comparison between the assay results of the developed RP-HPLC approach with those of the reported UPLC method [22] was done with regards to accuracy and precision respectively as shown in table 4. There was no significant difference between the proposed and the reported methods as the calculated values did not exceed the theoretical ones.

**Table 4.** Regression analysis results for determination of MET, LIN and EMP in spiked human plasma. (*a*, intercept; *b*, slope; *r*, correlation coefficient; *S*_a_, standard deviation of intercept; *S*_b_, standard deviation of slope; *S*_y/x_, residual standard deviation; LOD, limit of detection; LOQ, limit of quantitation.)

parameters	MET	EMP	LIN
concentration range (µg ml^−1^)	0.1–2	0.05–2	0.05–2
*r*	0.99998	0.99999	0.99998
*a*	0.006	7.60 × 10^−4^	0.022
*b*	0.446	0.436	1.142
*S*_a_	0.002	9.20 × 10^−4^	0.005
*S*_b_	0.002	8.92 × 10^−4^	0.004
*S*_(y/x)_	0.003	0.001	0.007
LOD	0.017	0.007	0.013
LOQ	0.052	0.021	0.040

### 3.5. Assay of the dosage form in Egypt

Dosage forms Empagliform^®^ and Gliptalina^®^ tablets containing MET with EMP and MET with LIN respectively, present in Egyptian markets in the ratios (500 : 12 : 5) and (500 : 2 : 5) respectively. Both tablets dosage forms were assayed using the proposed RP-HPLC method. Results are shown in the electronic supplementary material, table S7.

### 3.6. Results of spiked human plasma

Using the proposed method, three drugs, MET, LIN and EMP can be determined in spiked human plasma, as shown in figure 2*c*. Calibration curves were constructed for the three drugs by plotting the peak area against the drug concentration, covering the range of 0.05–2 µg ml^−1^ for both LIN and EMP and 0.1–2 µg ml^−1^ for MET. Table 4 shows the regression analysis results for the estimation of MET, LIN and EMP in spiked human plasma.

### 3.7. Results of greenness assessment

The sum of total penalty points for all procedures was calculated using the analytical eco-scale approach. The results reveal that the greenness of the proposed method was acceptable with an analytical eco-scale 73. The electronic supplementary material, table S8 shows the results of the greenness assessment.

## 4. Conclusion

The AQbD optimized and validated RP-HPLC technique was applied to identify and estimate MET, LIN, and EMP in their pure and laboratory-prepared tablet. It was extended to determine these drugs in spiked plasma samples. The RP-HPLC method could be an excellent candidate for quality control and bioanalytical analyses. A wide range of sample applications was upheld by the merits offered by AQbD methodology and MODR that helped lessen the number of OOT and OOS results by determining the design region where all stated ATP criteria were satisfied. Method robustness was proved by using factorial design along with the aid of MODR.

## Data Availability

Central Composite Design for AQbD design space determination: an HPLC method optimization for determination of Metformin, Linagliptin and Empagliflozin. Submitted with https://doi.org/10.5061/dryad.1ns1rn8w9 [45]. The data are provided in electronic supplementary material [46].
